# Spatial and temporal genetic structure of a river-resident Atlantic salmon (*Salmo salar*) after millennia of isolation

**DOI:** 10.1002/ece3.1040

**Published:** 2014-03-27

**Authors:** Odd Terje Sandlund, Sten Karlsson, Eva B Thorstad, Ole Kristian Berg, Matthew P Kent, Ine C J Norum, Kjetil Hindar

**Affiliations:** 1 Norwegian Institute for Nature Research (NINA)PO Box 5685, No-7485, Trondheim, Norway; 2 Department of Biology, Norwegian University of Science and Technology (NTNU)No-7491, Trondheim, Norway; 3 Department of Animal and Aquacultural Sciences (IHA), Center for Integrative Genetics (CIGENE), Norwegian University of Life SciencesPO Box 5003, No-1432, Ås, Norway

**Keywords:** Asymmetric gene flow, Atlantic salmon, habitat fragmentation, meta-population, river residency

## Abstract

The river-resident *Salmo salar* (“småblank”) has been isolated from other Atlantic salmon populations for 9,500 years in upper River Namsen, Norway. This is the only European Atlantic salmon population accomplishing its entire life cycle in a river. Hydropower development during the last six decades has introduced movement barriers and changed more than 50% of the river habitat to lentic conditions. Based on microsatellites and SNPs, genetic variation within småblank was only about 50% of that in the anadromous Atlantic salmon within the same river. The genetic differentiation (*F*_ST_) between småblank and the anadromous population was 0.24. This is similar to the differentiation between anadromous Atlantic salmon in Europe and North America. Microsatellite analyses identified three genetic subpopulations within småblank, each with an effective population size N_e_ of a few hundred individuals. There was no evidence of reduced heterozygosity and allelic richness in contemporary samples (2005–2008) compared with historical samples (1955–56 and 1978–79). However, there was a reduction in genetic differentiation between sampling localities over time. SNP data supported the differentiation of småblank into subpopulations and revealed downstream asymmetric gene flow between subpopulations. In spite of this, genetic variation was not higher in the lower than in the upper areas. The meta-population structure of småblank probably maintains genetic variation better than one panmictic population would do, as long as gene flow among subpopulations is maintained. Småblank is a unique endemic island population of Atlantic salmon. It is in a precarious situation due to a variety of anthropogenic impacts on its restricted habitat area. Thus, maintaining population size and avoiding further habitat fragmentation are important.

## Introduction

The understanding of genetic and structural processes in isolated populations is a central topic in conservation biology and genetics (e.g., Groom et al. 2006). During the history of life on Earth, geological processes have been important both in creating barriers between populations and in merging formerly isolated populations. Terrestrial and marine habitats are relatively continuous and may provide opportunities for gene flow among adjacent populations, in contrast to freshwater bodies, which are often discontinuous, and in many ways similar to islands. Many freshwater species therefore constitute a number of populations, which are isolated within restricted geographical areas (e.g., Brönmark and Hansson 2005). This low genetic connectivity has resulted in a large number of freshwater species (e.g., about 50% of teleost fishes; Wootton 1998) relative to the small amount of freshwater on Earth (about 2% of available water).

Fresh water bodies at higher latitudes harbor relatively few fish species. Since the last deglaciation, and the subsequent isostatic rebound, topography and climate have restricted immigration opportunities, resulting in species-poor fish communities. One major group entering freshwater systems in previously glaciated areas of both North America and Eurasia was the anadromous salmonids (e.g., Brönmark and Hansson 2005). In northwestern Europe, three salmonid fish species increased their distribution area in early postglacial times around 11,000 years ago, colonizing watercourses as they became accessible. Arctic charr (*Salvelinus alpinus* [L.]) and brown trout (*Salmo trutta* L.) readily established freshwater-resident populations, while Atlantic salmon (*Salmo salar* L.) rarely did so. Atlantic salmon normally maintain an anadromous life cycle, as all females smoltify (i.e., undergo physiologic and morphological changes to facilitate life in seawater) and migrate from the nursery stream to a more productive feeding environment in the sea, before returning to their nursery stream to spawn as adults. Some males remain in the river and mature sexually at a small body size (e.g., Webb et al. 2007; Jonsson and Jonsson 2011).

A few Atlantic salmon populations are freshwater stationary and use a freshwater lake for feeding and growing instead of migrating to the sea. This type of nonanadromous life cycle is common in parts of North America (Webb et al. 2007; Jonsson and Jonsson 2011), while only few populations are known in Europe. Eight localities with nonanadromous populations utilizing river–lake systems are known in Russia, one in Finland (Lake Saimaa area), one in Sweden (Lake Vänern), and two in Norway (River Otra/Lake Byglandsfjord and River Nidelva/Lake Nelaug, the latter being extinct) (Dahl 1928; Kazakov 1992; Nilsson et al. 2001; Barlaup et al. 2005; Säisä et al. 2005; Jonsson and Jonsson 2011). A completely river-resident life cycle is even more exceptional. Except watersheds in Newfoundland, Canada (Gibson et al. 1996; Webb et al. 2007), it is only known from River Namsen, Norway (Berg 1953, 1985). River-resident populations may arise when both sexes mature at the presmolt stage. Presmolt maturation is common in Atlantic salmon males, but rare in females (Hindar and Nordland 1989). Hence, river-resident populations have likely arisen from a low number of females and been subject to a strong founder effect. Being river resident also implies that all age-groups share a restricted area in fresh water. Anadromous salmon have the potential for larger population sizes because they utilize the sea during part of the life cycle. Anadromous salmon may also receive immigrants from other rivers (Jonsson and Jonsson 2011). Consequently, river-resident populations are expected to lose genetic variation at a higher rate.

**Figure 1:**
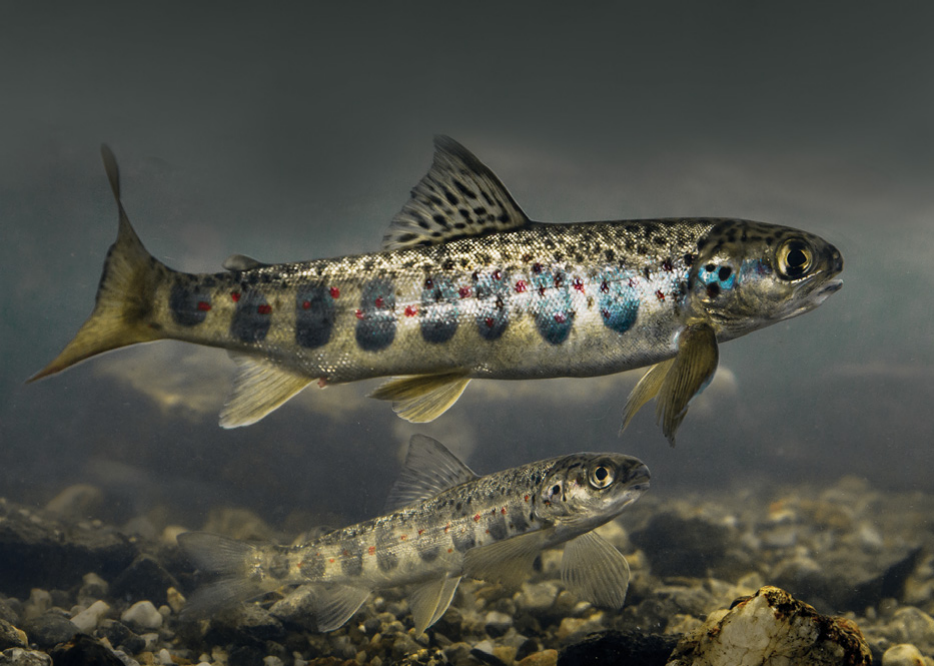
Live wild specimens of the river-resident Atlantic salmon (*Salmo salar*) “småblank” from River Namsen. Above is an adult female, veteran spawner, approximate body length and mass: 180 mm, 70 g. Below is a smaller adult male. Photograph: Per H. Olsen, NTNU, Trondheim, Norway.

The river-resident Atlantic salmon in River Namsen, Norway (Fig. 1; with the colloquial name “småblank,” which is used hereafter) constitutes a unique entity among European Atlantic salmon. Other freshwater-resident populations in Europe migrate to lakes, they have all been depleted, and most are presently maintained by stocking programmes (Ozerov et al. 2010; Jonsson and Jonsson 2011). The småblank in River Namsen constitutes an island population, which has been isolated for approximately 9,500 years (cf. Frankham 1997). Earlier analyses have demonstrated that småblank exhibits a lower genetic variation than the anadromous Atlantic salmon in the lower part of the river (Ståhl and Hindar 1988; Vuorinen and Berg 1989; Bourret et al. 2013). Allozymes analyses have also shown genetic differences between småblank populations (Vuorinen and Berg 1989). At the same time, there was no evidence of genetic signatures of offspring of anadromous Namsen salmon, which were released as fry in småblank territory during 1950–1976. By applying more fine-meshed methods (microsatellites and SNPs) to analyze samples from a number of localities within the restricted distribution area, and also to analyze samples collected over the last six decades, we aim to understand population viability and resilience of this unique Atlantic salmon population.

The hydromorphological features of the river sections inhabited by småblank indicate an asymmetric gene flow from the upper to the lower regions (Hänfling and Weetman 2006). Fish moving upstream will face more obstacles and barriers than fish moving downstream (Kawecki and Holt 2002). Asymmetric gene flow is a general phenomenon for many organisms living in habitats with predominantly unidirectional water currents (Pollux et al. 2009; Pringle et al. 2011), and indeed in most species with a source–sink metapopulation structure (Pulliam 1988; Hanski 1999). Over time, we may expect the upper subpopulations to lose genetic variation and that genetic variation is higher at the receiving end of the asymmetric gene flow (Hänfling and Weetman 2006). The småblank is threatened by habitat modifications due to hydropower development, causing severely reduced water flow and habitat fragmentation due to dams and weirs (Thorstad et al. 2009).

On this background, the following hypotheses were tested:

H1: The småblank population exhibits a fine-scale population structure with significant *F*_ST_ among subpopulations.

H2: Genetic variation is lower in the upper than in the lower region of the distribution area due to expected asymmetric gene flow.

H3: Over the last six decades, which is a period of severe habitat reduction and modification, småblank has experienced a substantial reduction in genetic variation.

## Material and Methods

### Study area

**Figure 2:**
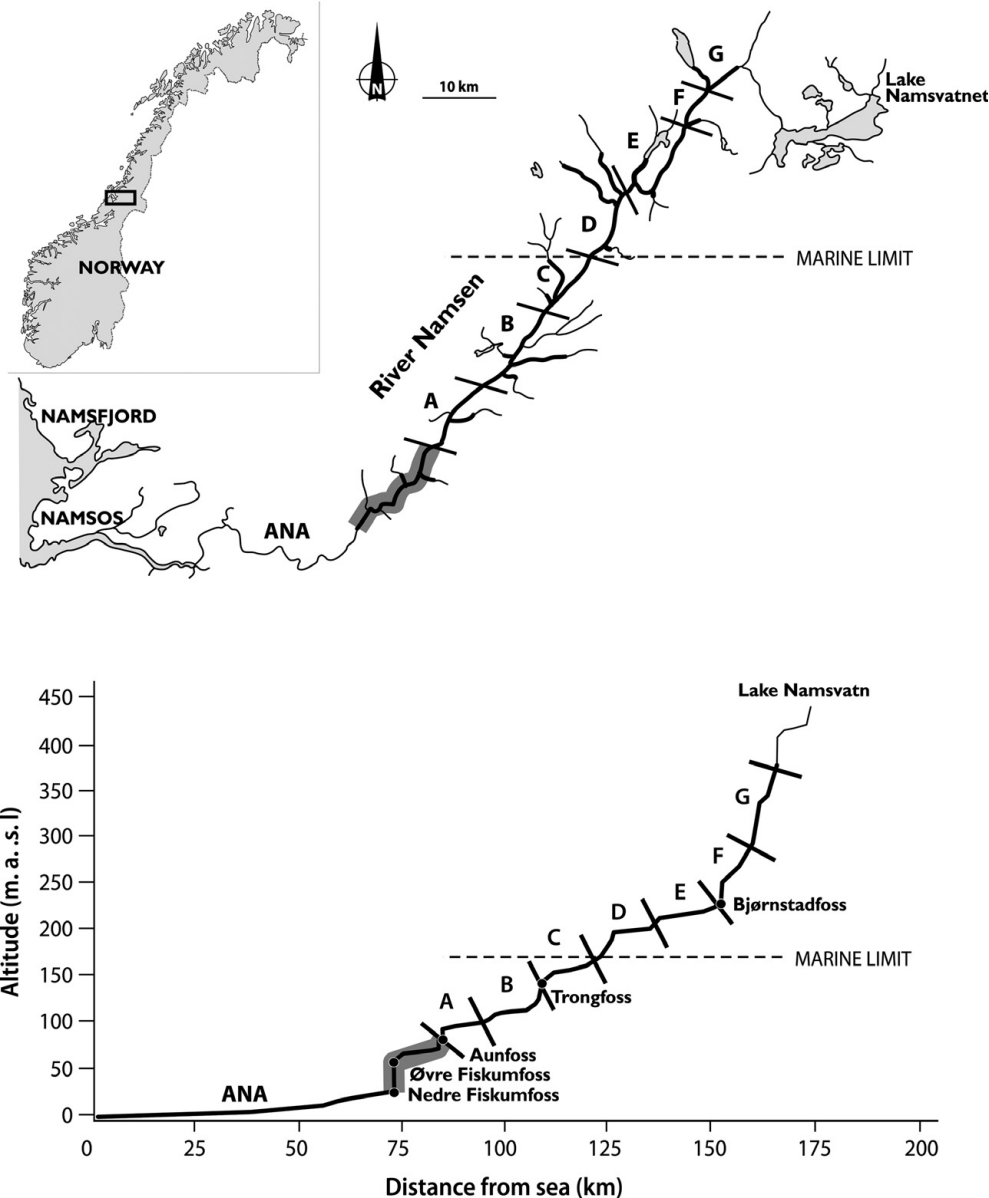
Location of River Namsen and the position of sampling localities A–G. Upper panel: map with the original distribution area of river-resident Atlantic salmon (“småblank”) in bold lines. The river section with overlapping småblank and anadromous Atlantic salmon is indicated by shading. Lower panel: schematic gradient of River Namsen. Symbols as in upper panel. Marine limit is the highest postglacial marine shoreline.

River Namsen runs for 210 km in Nord-Trøndelag County, central Norway, from Lake Namsvatnet (455 m a.s.l.) to the outlet in the sea at the town of Namsos (64.46°N, 11.51°E) (Fig. 2). The catchment area is 6,265 km^2^, and mean annual discharge at the outlet to the sea is 290 m^3^ sec^−1^. In the distribution area of småblank, the only other major fish species is brown trout, whereas three-spined stickleback (*Gasterosteus aculeatus* L.) occurs in the lower areas (A and B, Fig. 2; Berg 1984). European minnow (*Phoxinus phoxinus* L.) has been introduced to the upper parts of the river system and is spreading downstream, but has not yet reached the småblank areas (own unpublished data).

### Sampling of landlocked salmon (småblank)

Male småblank mature sexually between 2 and 4 years, and females between 3 and 5 years. The smallest mature males observed were 120 mm long (total length), while the smallest mature females were 175 mm (Berg and Gausen 1988; Thorstad et al. 2009; Norum 2010). The largest reported individual was 295 mm long.

Fin-clip samples for genetic analyses were collected between 2005 and 2008 from fish caught in six areas in the main river (Fig. 2, localities A–F) and from the tributary River Mellingselva (Fig. 2, locality G) (Table 1). Within each area, fish were collected from several locations in an effort to decrease relatedness between individuals. In order to analyze the temporal stability of the genetic structure, genetic analyses were also performed on scale samples collected between 1955–1956 and 1978–1979 (in total 100 fish) from three river sections (Table 1; identified with the subscripts 55, 56, 78, and 79). Scales from anadromous Atlantic salmon, collected in the lower sections of River Namsen in 1978 (Table 1, locality ANA_78_, cf. river section ANA in Fig. 2), were analyzed in order to compare småblank and anadromous salmon from the same river. In total, samples from 312 individual fish were subject to genetic analyses. The reason for analyzing samples of anadromous salmon from 1978 was that they were collected before the start of salmon aquaculture. Present-day samples may have been influenced by introgression with escaped farmed salmon from coastal aquaculture (Glover et al. 2013).

**Table 1:** Localities, sampling years, and number of samples for genetic analyses of anadromous and river-resident Atlantic salmon (småblank) in River Namsen and the tributary River Mellingselva. Sampling localities are indicated in Fig. 2 by their letter symbols: A–G and ANA. Recent samples (2005–2008) are fin clips, older samples are scales. Geographical positions of sections are given in decimal degrees from downstream to upstream end of actual sampling locality

Sampling locality		Position (decimal degrees)	Year	# fish	Distance from sea (km)	Elevation (m a.s.l.)
ANA_78_	River Namsen, anadromous section	64,46748°N 11,54462°E- 64,54188°N 12,45527°E	1978	35	0–83	0
A	Dam Aunfoss to dam Åsmulfoss	64,61126°N 12,57973°E- 64,67698°N 12,67656°E	2005–08	14	84–93	94
A_79_		As above	1979	26		
B	Dam Åsmulfoss to Trongfoss	64,67698°N 12,67656°E- 64,75004°N 12,84708°E	2005–08	16	94–107	101
C	Trongfoss to weir Kjelmyrfoss	64,75004°N 12,84708°E- 64,81540°N 12,96043°E	2008	53	108–116	144
D	Weir Kjelmyrfoss to below weir Bjørhusdal	64,81540°N 12,96043°E- 64,89635°N 13,07082°E	2005–08	17	117–129	158
E	Weir Namsskogan to below Bjørnstadfoss	64,93152°N 13,16340°E- 65,01149°N 13,26039°E	2005–08	26	137–147	210
E_78_		65,01149°N 13,26039°E	1978	30		
E_55_		65,01149°N 13,26039°E	1955	15		
F	Above Bjørnstadfoss to outlet River Mellingselva	65,01555°N 13,25750°E- 65,05575°N 13,31968°E	2005–08	28	148–153	221
G	River Mellingselva	65,05575°N 13,31968°E- 65,07937°N 13,28596°E	2008	23	154	246
G_78_		As above	1978	19		
G_56_		As above	1956	10		

### Distribution and habitat

The habitat area estimates for småblank were based on aerial photos from 3 June 2003 to 22 July 2006 (Thorstad et al. 2009) and later field classification of habitat quality (Norum 2010). Areas were determined using the GIS-program ArcMap 9.3.

The original distribution of småblank included the main river from above the waterfall Nedre Fiskumfoss (elevation top 58 m a.s.l.) up to Namskroken (286 m a.s.l.), a distance of about 85 km (Berg 1953; Thorstad et al. 2009; Fig. 2). Construction of hydropower dams and fish passes has allowed access for anadromous Atlantic salmon up to the waterfall Aunfoss (95 m a.s.l.), reducing the allopatric distribution of småblank by 10 km in the main river. We do not know the status of småblank in the area where it is sympatric with anadromous salmon, and whether småblank and anadromous salmon hybridize. Today, the hydropower dam at Aunfoss separates anadromous salmon and småblank in the main river so that småblank are allopatric in 75 km of the main river (Fig. 2, localities A–G) between Aunfoss and Namskroken. Along this river section, småblank also occur in the lower reaches of a number of tributaries (Fig. 2). The total water-covered area where allopatric småblank have been recorded in River Namsen and tributaries amounts to 12.5 km^2^, with the major proportion (87%) in the main river.

The river gradient along the småblank distribution area (dammed areas excluded) varies between 0.8 and 12 m km^−1^ (Fig. 2). There are several waterfalls, but only Trongfoss (cf. Fig. 2), with a vertical fall of more than 10 m, is considered to be a complete barrier to upstream movement. The smaller waterfall Bjørnstadfoss (cf. Fig. 2) is probably passable for upstream movement at certain water discharges. The construction of Åsmulfoss dam and hydropower station (around 1970) created a permanent barrier where only a number of small rapids occurred before. Since 1970, weirs have been constructed at several sites, acting either as permanent barriers or obstacles at certain water discharges. Both dams and weirs cause hydromorphological changes promoting lentic instead of lotic conditions. In total, 38 km of the 75-km-long river stretch (51%) available for småblank in the main river **ar**e presently affected by the change to a more lentic habitat. In addition to habitat degradation, water has been diverted from the main river for hydro-electric power production. The effect of this water diversion is that the size of the remaining catchment area upstream of Bjørnstadfoss (147 km from the sea; Fig. 1) is presently only 1/3 of the natural situation. As a consequence, annual water discharge and water velocities are reduced.

### Genetic methods

The fish material was assayed for genetic variation at eight microsatellite loci, and a subsample of 88 individuals of småblank from three sampling localities was also assayed for genetic variation at 4414 SNP loci (Table 2 and Fig. 2). The SNP data were available from a previous study (Bourret et al. 2013). Although these data concern fewer localities and specimens, they were included here to add extra statistical power to the analyses. DNA was extracted from ethanol preserved fin clips and scales using the E.Z.N.A™ tissue DNA kit (E.Z.N.A.® Omega Bio-Tek Inc, Norcross, GA).

**Table 2:** Summary statistics of eight microsatellite loci and 4414 SNP loci from the anadromous and river-resident (småblank) Atlantic salmon in River Namsen. *N* is sample size, H_e_ is expected heterozygosity, H_o_ is observed heterozygosity, #A is observed numbers of different alleles, A_R_ is allelic richness based on five diploid individuals, P_H–W_ is probability of conformance to Hardy–Weinberg expectation, %P is proportion of polymorphic SNP loci, A_E_ is average effective number of alleles at SNP loci. Sampling localities are indicated in Fig. 2

	Microsatellite loci					
	
Sampling locality	*N*	H_e_	H_o_	#A	A_R_	P_H–W_
ANA_78_	35	0.722	0.717	9.6	4.9	0.2802
A	14	0.359	0.304	2.9	2.3	0.3436
B	16	0.346	0.344	2.9	2.2	0.9204
C	53	0.377	0.346	3.5	2.4	**0.0042**
D	17	0.380	0.412	3.5	2.5	0.5557
E	26	0.370	0.418	3.8	2.4	0.1325
F	28	0.361	0.375	3.9	2.6	0.2479
G	23	0.320	0.315	3.4	2.3	**0.0016**
E_55_	15	0.344	0.457	2.8	2.4	**0.0038**
E_78_	30	0.355	0.366	3.3	2.5	0.9849
G_56_	10	0.305	0.303	2.3	2.1	0.9544
G_78_	19	0.360	0.322	3.1	2.5	0.1795
A_79_	26	0.321	0.298	3.0	2.2	0.2056

	SNP loci					
	
Sampling locality	*N*	H_e_	H_o_	%P	A_E_	P_H–W_
ANA_78_	48	0.345	0.342	96.7	1.591	**∼0**
B	16	0.126	0.127	40.8	1.213	∼1
C	11	0.120	0.127	35.8	1.204	∼1
E	13	0.128	0.130	37.5	1.217	∼1

Significant *P*-values are in bold.

PCR was carried out in two different multiplexes containing the following microsatellite loci: Ssa289, Ssa14 (McConnell et al. 1995), Ssa171, Ssa197 (O'Reilly et al. 1996), Ssa408 (Cairney et al. 2000) in multiplex 1, *μ*20.19 (Sanchez et al. 1996), Ssosl85 (Slettan et al. 1995), Ssosl438 (Slettan et al. 1996) in multiplex 2. The multiplex reactions were carried out in a total volume of 10 *μ*L, containing 11 *μ*mol/L and 6.5 *μ*mol/L of total primers, in multiplex 1 and 2, respectively, but with different concentrations for each pair, 1 mmol/L of total dNTP, 1X reaction buffer, 2.25 mmol/L MgCl_2_, and 0.75 units of Thermostart taq polymerase (Thermo Scientific Inc., Waltham, MA). The following PCR program was run on a Quattro Cycler (VWR): denaturation for 15 min at 95°C: six cycles touchdown PCR of denaturation at 94°C, annealing temperature from 58°C to 52 °C, and extension at 72°C for 60 s. The last 24 cycles were run with denaturation at 94°C for 30 s, annealing at 52°C for 30 s, and extension at 72°C for 60 s; a final step of extension at 72°C for 10 min. Fragments from each multiplex were separated and visualized separately on an ABI 3130xl DNA analyser (Applied Biosystems) and sizing using genemapper ver. 3.7 (Applied Biosystems, Foster City, CA).

SNP genotype data were obtained from two different projects. The SNP genotype data for the anadromous Atlantic salmon from River Namsen were described by Karlsson et al. (2011), using the 7K Atlantic salmon SNP chip (CIGENE, Ås, Norway). The SNP chip data for the småblank salmon from River Namsen watercourse were described by Bourret et al. (2013), also using the 7K Atlantic salmon SNP chip (CIGENE). The two datasets were combined, and a common set of 4414 SNPs was used in this study.

Test of conformity to Hardy–Weinberg equilibrium, estimates of observed and expected heterozygosity, homogeneity test of allele frequencies, and *F*_ST_ estimates according to Weir and Cockerham (1984) were carried out in genepop v.4 (Raymond and Rousset 1995), for microsatellite data and SNP data. Number of alleles and number of alleles independent of sample size (allelic richness) were estimated for the microsatellite data using Fstat v. 2.9.3 (Goudet 2001). Proportion of polymorphic loci and effective number of alleles were estimated for the SNP data using genalex (Peakall and Smouse 2006).

Possible differences in allelic richness, expected heterozygosity, and effective number of alleles between sampling localities were tested using Wilcoxon signed-ranked test as implemented in SPSS Statistics 18 (http://www.spss.com/). Possible differences in proportion of polymorphic loci (SNPs) were tested by a random resampling approach, using POP tools (an add-in program in excel available at: http://www.poptools.org/). Random sampling was performed 1,000 times, and for each sampling, the proportion of polymorphic loci was estimated. The average proportion of polymorphic loci from each random sampling and their upper (0.975) and lower (0.025) percentiles were recorded and compared with the observed proportion of polymorphic loci in the sampling locality for which there were as many individuals as were resampled in the locality with a larger sample size.

Population structure in the småblank was explored from pairwise estimates of *F*_ST_, analysis of molecular variance (AMOVA) implemented in arlequin ver. 3.5.1.2 (Schneider et al. 2000), analysis of individual probability of belonging to different number of populations without a priori information of sampling locality using STRUCTURE ver. 2.3.1 (Pritchard et al. 2000), and from pairwise estimates of genetic distance according to Nei (1972, 1978) using Phylip ver. 3.69 (Felsenstein 2009). In STRUCTURE, individuals were probabilistically assigned to a predefined number of populations, ranging from one (K = 1) to twelve (K = 12), without a priori information of sampling locality while applying the admixture model. Each run was repeated three times with 50,000 repetitions as burn-in and 100,000 repetitions after burn-in. The STRUCTURE HARVESTER program (Earl and von Holdt 2012) was used for estimating the most likely number of populations using the Delta_K method (Evanno et al. 2005) and the Ln probability of the data estimates (Pritchard et al. 2000).

Putative first-generation migrants were detected from using STRUCTURE ver. 2.3.1 (Pritchard et al. 2000) and from individual self-assignment using the Bayesian method (Rannala and Mountain 1997) implemented in GeneClass2 (Piry et al. 2004). For the latter, the individuals to be assigned were all included in the reference populations to which the populations were assigned (self-assignment). Genetic self-assignment was also used for detecting possible signatures of genetic introgression from anadromous salmon in the småblank samples.

Effective population size was estimated using the temporal method by Jorde and Ryman (2007), with the Fs estimator implemented in the TempoFS software (Jorde and Ryman 2007) available at http://www.zoologi.su.se/∼ryman/ and the maximum-likelihood method by Wang and Whitlock (2003), implemented in the MNe 1.0 program available at http://www.zsl.org/science/research-projects/software/mlne,1151,AR.html. With the latter program, migration rates were estimated using all available samples as sources for immigrants. Estimates of effective population size using the temporal methods require a minimum of two temporal samples from the same population. In this study, temporal samples were available from three sampling localities. For the remaining samples (and the temporal samples), effective population size was estimated using the linkage disequilibrium method (LD) by Hill (1981) with the bias correction for sample sizes (Waples 2006), as implemented in the LDNe program by Waples and Do (2008). The demographic history of the populations was examined using the M-test by Garza and Williamson (2001). The M-value is the ratio of the number of alleles and the allele size range at microsatellite loci. A population that has recently experienced a reduction in effective population size is expected to show a lower M-value, because the number of alleles is expected to decline faster than the range in allele size, under a step-wise mutation model (smm) (Garza and Williamson 2001). All microsatellite loci appeared to fit the assumption of a SMM model, except for one locus (Ssa171) with alleles separated by both four and two base pairs. To determine whether the estimated M ratio represented a signature of a recent decline in population size, the conservative critical M-value of 0.68 was applied, as proposed by Garza and Williamson (2001).

### Genetic data

Of the 312 individuals assayed for genetic variation at eight microsatellite loci, 190 had a scoring rate of 100%. Genotypes were missing at one locus for 70 individuals, at two loci for 27 individuals, at three loci for 14 individuals, at four loci for six individuals, at five loci for three individuals, and at six loci for two individuals. Significant deviation from Hardy–Weinberg equilibrium was detected in three samples: C, G, and E_55_ (Table 2). A closer examination of deviation from Hardy–Weinberg equilibrium at individual loci and samples detected no consistent trends in deficits or excess of heterozygotes (Appendix 1). All microsatellite loci were used in the further analyses.

Of the 88 individuals assayed for genetic variation at 4414 SNP loci from the locations B, C, and E, 79 individuals had a scoring rate larger than 95%, and all individuals had a scoring rate equal to or higher than 90%. Significant deviation from Hardy–Weinberg equilibrium was detected in the samples from the anadromous population but not in the småblank samples (Table 2). In the anadromous population, 3.7% of the informative loci had a probability < 0.05 of being in Hardy–Weinberg equilibrium. In the B, C, and E småblank samples, 4.9, 1.6, and 3.3% of the informative loci had probabilities < 0.05 of being in Hardy–Weinberg equilibrium, respectively. All SNP loci were used in the further analyses.

## Results

### Småblank vs. anadromous Atlantic salmon

#### Microsatellites

Expected heterozygosity and allelic richness within småblank sampling locations were much lower (*P* < 0.012 for each pairwise comparison, Wilcoxon signed-rank test) than the anadromous population (Table 2). Expected heterozygosity in the småblank samples ranged from 0.31 to 0.38, while expected heterozygosity in the anadromous population was 0.72. Allelic richness ranged from 2.1 to 2.6 in the småblank samples and was 4.9 in the anadromous population. Two loci (Ssa14 and *μ*20.19) were monomorphic in all småblank samples, but polymorphic in the anadromous population, with two and three alleles, respectively (Appendix 2). Pairwise estimates of genetic differentiation (*F*_ST_) between the anadromous population and the småblank samples ranged from 0.22 to 0.26 and were significantly larger than zero (P ≈ 0). There were no signatures of genetic introgression from anadromous salmon in the småblank samples as none of the småblank specimens assigned with a relative assignment score higher than 0.02% to the anadromous sample, whereas all specimens had an assignment score ∼100% to the småblank samples.

#### SNPs

In agreement with the microsatellite data, the anadromous population had about twice as much genetic variation as the småblank samples based on the SNP data (Table 2). The expected heterozygosity ranged from 0.12 to 0.13 in the three småblank sampling localities (B, C, E) and was 0.34 in the anadromous population (P ≈ 0 for each pairwise comparison, Wilcoxon signed-rank test). The anadromous population was polymorphic for 96.6% of the loci, while the småblank samples were polymorphic for only 35.8–40.8% of the loci. Random sampling (100 times) of 13 individuals from the anadromous population resulted in a mean proportion of polymorphic loci of 0.91 with a 95% confidence interval of 0.88–0.92, which is thus highly significant.

Pairwise *F*_ST_ estimates between the anadromous population and the B, C, and E småblank samples were 0.28, 0.27, and 0.27, respectively (P ≈ 0). In agreement with the results from microsatellite loci, no signatures of genetic introgression from anadromous salmon were found in the småblank samples, as all småblank specimens had a relative assignment score of zero to the anadromous sample, but 100% to the småblank samples.

### Genetic structure of the småblank

#### Microsatellites

Average expected heterozygosity (range: 0.305–0.377) and allelic richness (range: 2.1–2.6) did not differ significantly between pairs of småblank sample localities (Wilcoxon signed-rank test).

From pairwise *F*_ST_ estimates and homogeneity tests between pairs of sampling locations (Appendix 2), two main groups appeared: Group I comprising the A, B, C, and D localities, and Group II comprising the F and G localities. The samples from the E locality were most closely associated with Group II in 1955–1978 and with Group I in 2005–2008. The genetic structure was further explored with an AMOVA, whereby the E and the E_55_/E_78_ samples were placed in separate groups or in the two main groups, respectively. The largest genetic variation among groups (*F*_CT_) was obtained when the E_55_/E_78_ samples were placed in Group II and the E sample placed in a separate group (*F*_CT_ = 0.081, P∼0). When the E_55_/E_78_ samples and the E sample were placed in separate groups, the among-group variance component was 0.073 (P∼0). When the E_55_/E_78_ samples and the E sample were both placed in Group II, the variance among groups was relatively high (*F*_CT_ = 0.080, *P* = 0.001), but the variance among populations within group was larger and significant (*P* = 0.042). From these analyses, there appears to be at least three genetic clusters: one in the lower region of the distribution area, represented by the samples from A, B, C, and D, one in the uppermost region, represented by the samples from F and G, and one in between the lower and upper region, represented by the E samples.

**Figure 3:**
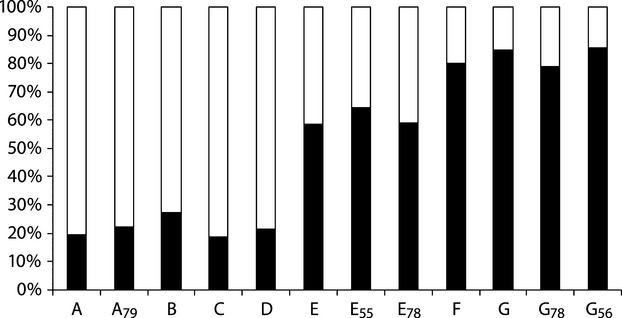
Average proportion of genome membership, from genotypes at eight microsatellite loci for individuals of river-resident Atlantic salmon (småblank) sampled at eight localities in River Namsen, assuming two populations (*K* = 2) and applying the admixture model in STRUCTURE. Sampling localities are A–G (cf. Fig. 2). For older samples, sampling years are given as subscript.

The population structure of the småblank samples was further explored by individual genetic assignment to a predefined number of populations (K) using STRUCTURE. The most likely number of populations was two according to the Delta-K value (Delta-K = 25.42) and the Ln probability of the data (Mean LnP(K) = −2805.40), represented by the A, B, C, and D samples in one cluster, the F and G samples in the second cluster, and the E samples positioned in between the two clusters (Fig. 3).

**Figure 4:**
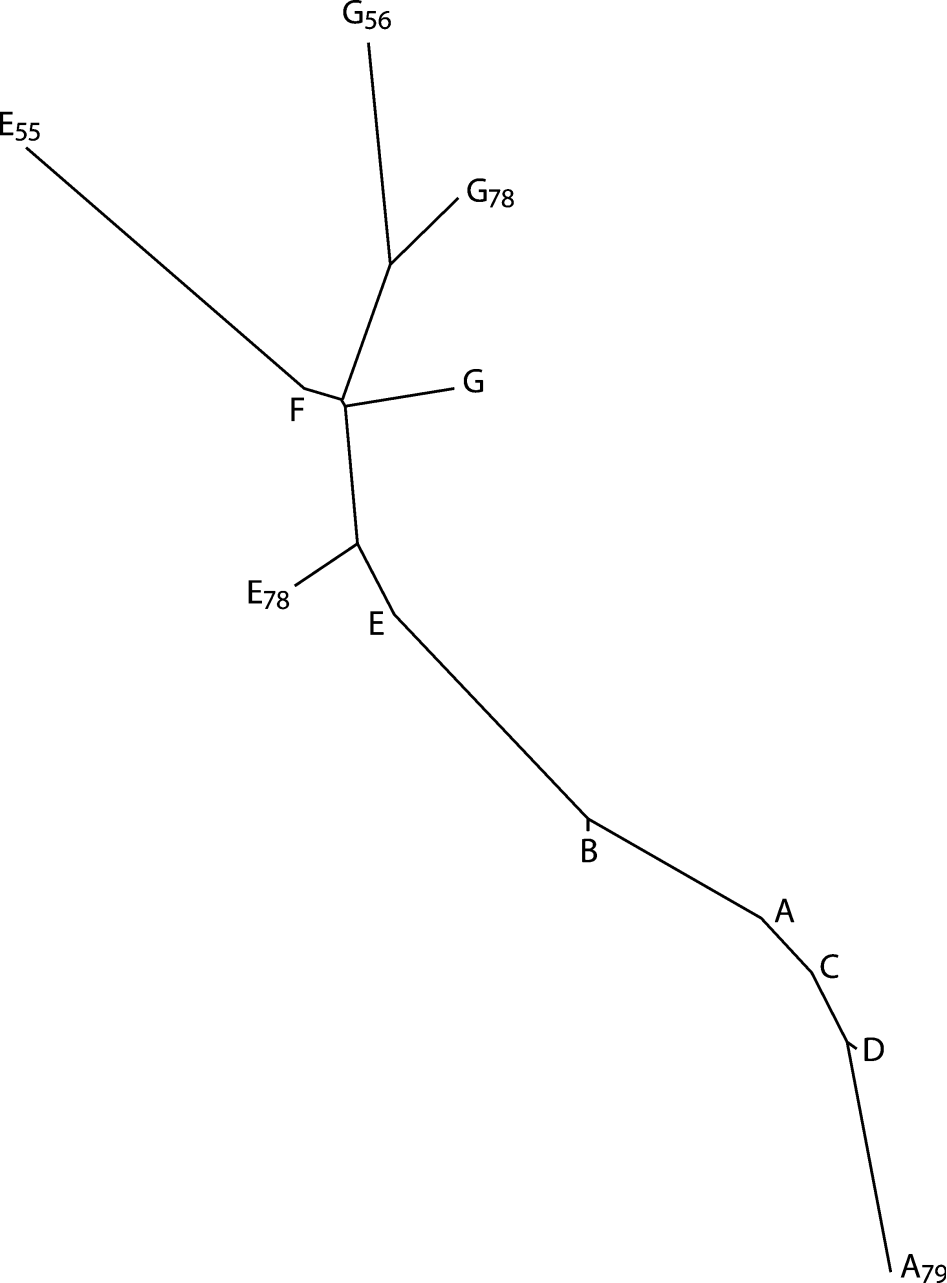
Neighbor-joining dendrogram generated from pairwise estimates of Nei's genetic distance from samples of river-resident Atlantic salmon (småblank) in River Namsen watercourse from eight sampling localities, including temporal samples, using eight microsatellite loci. Sampling localities are shown in Table 1 and Fig. 2.

The genetic differences among the småblank sampling localities are summarized in a neighbor-joining dendrogram (Fig. 4). The two main clusters, represented by samples, form the lower and upper regions of the river are visible, and the E sample is positioned in between these two clusters. The sample collected at Bjørnstad in 1955 (E_55_), clustered with the samples from the upper region, while the samples collected in 1978 (E_78_) clustered somewhat in between the two main clusters.

#### SNPs

There were no significant differences in the effective number of alleles, expected heterozygosity (Wilcoxon signed-rank test) or in the proportion of polymorphic loci (random resampling) between pairs of sampling localities (Table 2).

**Figure 5:**
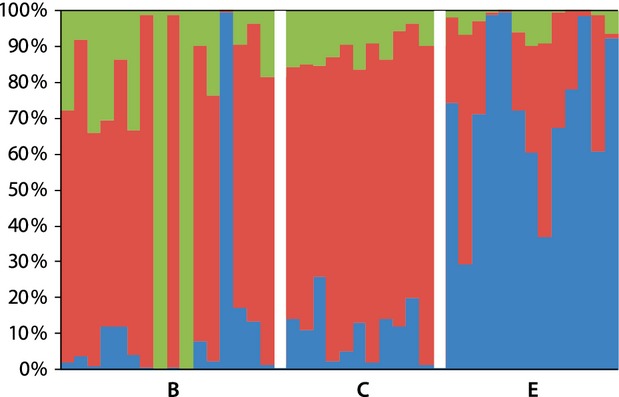
Proportion of genome membership for each individual of river-resident Atlantic salmon (småblank) sampled at three localities (B, C, and E, separated by blank bars; cf. Fig. 2), in River Namsen genotyped at 4414 SNP loci, assuming three populations (*K* = 3), represented by three different colors.

The genetic differences indicated by microsatellites, between the E sample and the samples from A, B, C, and D, were supported by the SNP data with an *F*_ST_ of 0.046 (P∼0) between the E and B samples, and 0.053 (P∼0) between the E and C samples. In agreement with the microsatellite data, the SNP analysis showed no significant difference in allele frequencies between B and C (*F*_ST_ = 0.027, *P* = 0.99), and the most likely number of populations detected by STRUCTURE was two according to the Delta-K value (Delta-K = 166.52) but three according to the Ln probability of the data (Mean LnP(K) = −68850). From a closer examination of the results from the STRUCTURE analyses, assuming three populations, (Fig. 5) one population was represented by the individuals from E, one population was represented by individuals from B and C, and one population was represented by only two individuals from B. These two individuals had a probability of 0.998 of belonging to the third population, which otherwise was not represented in our samples. Another individual from B had a probability of 0.995 of belonging to the population represented by the E samples. The STRUCTURE results were almost identical when assuming two instead of three populations. The exception was that when assuming two populations, the two individuals from location B representing the third population (K = 3) were assigned to the same populations as the individuals from the downstream locations B and C. From self-assignment using GeneClass, the one individual from B was assigned to the E samples (relative log-likelihood score = 100%), in agreement with STRUCTURE, while the other two individuals from the B locality with unknown origin from the STRUCTURE analysis were assigned to the B locality from where they were sampled (relative log-likelihood score = 100%). These observations suggest that there may be three (18.8%) first-generation migrants among the individuals sampled at the B locality, two for which the populations are not included in the SNP dataset, and one with a genetic signature matching the E samples.

### Temporal genetic variation in småblank

Historical samples from the A, E, and G sampling localities were analyzed for genetic variation at the eight microsatellite loci (Appendix 1). Average expected heterozygosity and allelic richness (Table 2) did not differ significantly between historical samples and contemporary samples within sampling localities (Wilcoxon signed-rank test). No significant temporal instability in allele frequencies (Appendix 2) was observed between the samples from 1979 and 2005–2008 at the A locality (*F*_ST_ = −0.01, *P* = 0.94), nor between samples from 1956, 1978, and 2008 at the G locality (G_56_ vs. G: *F*_ST_ = 0.032, *P* = 0.114; G vs. G_78_: *F*_ST_ = 0.011, *P* = 0.244; G_78_ vs. G_56_: *F*_ST_ = 0.034, *P* = 0.059). However, between the E samples from 1955 and 1978, a significant temporal instability in allele frequencies was observed (*F*_ST_ = 0.04, *P* = 0.002).

The impression of a general trend of decreasing genetic differentiation among localities over time emerging from Fig. 2 was specifically tested for pairs of localities where temporal samples were available, that is, between A and E, A and G, and E and G (cf. Fig. 2). The genetic difference between the A and E was highly significant (*P* = 0.000) in 1979/1978 and significant (*P* = 0.020) in 2005/2008, and the estimated *F*_ST_ was higher in 1978/1979 (*F*_ST_ = 0.084) compared with the 2005/2008 samples (*F*_ST_ = 0.018). The genetic difference between the A and G localities was highly significant (*P* = 0.000) in 1979/1978 and also in 2005/2008, but the *F*_ST_ estimate was somewhat higher in 1978/1979 (*F*_ST_ = 0.112) than in 2005/2008 (*F*_ST_ = 0.077). The genetic difference between the E and G localities was higher and significant in the 1955/1956 samples (*F*_ST_ = 0.051, *P* = 0.001), compared with the nonsignificant 1978 samples (*F*_ST_ = 0.019, *P* = 0.076). The 2005/2008 samples from the E and G localities were significantly different (*P* = 0.021), but with an *F*_ST_ estimate (*F*_ST_ = 0.02) not significantly different from 1978. Jack-knifing over five polymorphic loci demonstrated that the latter test had low power (i.e., large estimated standard errors).

### Effective population size and migration rate

Based on the temporal methods, effective småblank population size (N_e_) ranged from 29 (E locality) to 302 (G locality), with large confidence intervals for some estimates (Table 3). The estimate of migration rates was 0.038 in the G locality, while increasing further downstream in the E locality (0.12) and the A locality (0.17), with large confidence intervals (Table 3). The linkage disequilibrium method gave poor results based on microsatellite data, with negative estimates and very large or infinite confidence intervals. Based on SNP data, N_e_ was 268 in the anadromous sample, while N_e_ for småblank was 28 at the B and C localities combined, and 619 for the E locality (Table 3). The larger N_e_ estimate for the E locality compared with the anadromous samples might reflect a possible bias (Waples and Do 2010) and represent the effective population size of the metapopulation including localities E, F, and G (c.f. Gomez-Uchida et al. 2013). All småblank samples had significantly lower M-values than the conservative critical M-value (0.68) proposed by Garza and Williamson (2001). The average M-value for the anadromous population was 0.87 using seven informative microsatellite loci (range: 0.55–1.0). For the småblank samples, there were five informative loci. The average M-value for the pooled contemporary samples from A, B, C, and D was 0.54 (range: 0.3–0.70). For the E sample, the average M-value was 0.49 (range: 0.22–0.75). The average M-value for the pooled contemporary samples from F and G was 0.55 (range: 0.33–0.8). M-values for the historical samples did not appear to differ from the contemporary samples, with M = 0.53 for the E_55_/E_78_ samples and 0.42 for the A_79_ sample. The low M-values in småblank should not immediately be interpreted as a sign of a recent bottleneck because the M-values might be biased from a few outlier alleles (especially in the Ssosl438 and Ssosl85 loci) and because only five loci were examined. Furthermore, the number and origin of founders, the long-term effective population size (N_e_), and possible fluctuations in N_e_ are not known, which makes it difficult to deduce whether the M-values are due to recent or past demographic events (Garza and Williamson 2001).

**Table 3:** Estimates of effective population size (Ne) using temporal methods (TempoFS and MlNe) and the linkage disequilibrium method (LDNe) for samples of anadromous and river-resident (småblank) Atlantic salmon in River Namsen. Estimates of migration rates and Ne were jointly obtained from the temporal method by Wang and Whitlock (2003). One dataset with eight microsatellite loci and one dataset with 4414 SNP loci were used as indicated in the table (Marker type)

Temporal method – Tempo FS			
Pop	Ne (95% CI)		Marker type
E_55_–E_78_	29 (16–168)		Msat
G_56_–G_78_	261 (28–∞)		Msat
G_78_–G	88 (27–∞)		Msat
G_56_–G	126 (35–∞)		Msat
A_79_–A	∞ (178–∞)		Msat

Temporal method – MlNe			
Pop	Ne (95% CI)	m (95% CI)	Marker type
E_55_–E_78_	36 (14–176)	0.12 (0.042–0.35)	Msat
G_56_–G_78_	30 (9–∞)	0.99 (0.09–∞)	Msat
G_78_–G	302 (54–∞)	0.006 (0.001–0.045)	Msat
G_56_–G	79 (28–373)	0.038 (0.0062–0.1)	Msat
A_79_–A	213 (77–∞)	0.17 (∞–∞)	Msat

Moment estimate – LDNe			
Pop	Ne (95% CI)		Marker type
ANA_78_	∞ (141–∞)		Msat
A, B, C, D	266 (54–∞)		Msat
E	∞ (106–∞)		Msat
F, G	58 (22–3216)		Msat
ANA_78_	268 (263–274)		SNPs
B, C	28 (27–28)		SNPs
E	619 (406–1295)		SNPs

## Discussion

The heterozygosity and allelic richness of the river-resident småblank was about 50% lower than that of the anadromous Atlantic salmon in the lower river sections. We found no signature of genetic introgression from releases of offspring of anadromous salmon in the småblank samples. These findings confirm earlier observations in enzyme-coding genes for småblank (Vuorinen and Berg 1989). Småblank have been isolated from other conspecific populations for approximately 9,500 years (Berg 1985), corresponding to some 2000 generations. It is reasonable to assume that a major reduction in genetic variation was caused by founder effects and genetic drift during the first generations after isolation. The processes when småblank became isolated from anadromous Atlantic salmon several thousand years ago are not known, but the low frequency of mature resident females in anadromous populations (Hindar and Nordland 1989) indicates that the founding event may have involved only a few resident females. Genetic drift and other stochastic processes in restricted populations may have caused the low genetic diversity indicated by the low heterozygosity in småblank as well as among other landlocked populations compared with anadromous populations (Vuorinen and Berg 1989; Ozerov et al. 2012; Bourret et al. 2013; Perrier et al. 2013). The level of genetic variation in the småblank (island) population relative to the anadromous salmon (mainland) population is similar to island/mainland pairs in other taxa that have been separated for a similar time period (e.g., Frankham 1997; Hinten et al. 2003; Whiteley et al. 2010).

A large-scale genetic comparison based on SNPs of 38 Atlantic salmon populations in Europe and North America revealed that nonanadromous populations represented distinct outliers in all regions (Bourret et al. 2013). Among anadromous populations, differentiation between European and North American populations accounted for 41% of variation, whereas within Europe, three major groups (Atlantic, Baltic, and Barents–White Sea) accounted for 8% of the variation. Småblank clustered differently from anadromous salmon of European origin and, genetically, was no more closely related to Norwegian than to other European anadromous populations (Fig. 2 and Table S1 in Bourret et al. 2013). Based on the 4414 SNP loci used in the present study, estimated *F*_ST_ between the småblank samples and the anadromous population in River Namsen ranged from 0.27 to 0.28, while estimated *F*_ST_ among 13 Norwegian anadromous populations using the same SNP loci was 0.038 (Karlsson et al. 2011). Based on microsatellite loci, the genetic distance between småblank and anadromous Atlantic salmon in River Namsen was estimated at *F*_ST_ = 0.24, which is similar to the difference between anadromous Atlantic salmon in Europe and North America (Ståhl 1987; King et al. 2007).

Our results indicate that småblank is subdivided into three genetically different populations. One population occupies the habitat from Aunfoss up to the weir at Kjelmyrfoss (section A-D, a river section of more than 30 km). The second population inhabits the river section from the weir at Namsskogan up to the Bjørnstadfoss waterfall (section E, approximately 10 km). The third population occupies the main river from the Bjørnstadfoss waterfall up to and including the tributary River Mellingselva (section F and G, a little more than 10 km). The subdivision of småblank in several populations based on samples from 2005–2008 was also indicated in the samples from the 1950s and 1970s. Similarly, enzyme electrophoresis performed in the 1980s indicated a subdivision of the småblank in two different genetic clusters corresponding to the upper and lower regions of the distribution (Vuorinen and Berg 1989). This subdivision is maintained within a limited habitat area of 12.5 km^2^, and it is likely determined by obstacles to upstream movement. The genetic differentiation is to some extent reflected in life-history differentiation. Body size at maturity is smaller in fish sampled in the upper area (localities F and G), compared with other localities (Berg and Gausen 1988; Thorstad et al. 2009). The morphology of subpopulations has not been investigated.

Effective population size estimates indicate N_e_ values at a few thousand småblank in total. Assuming that the division in subpopulations is a persistent pattern, each subpopulation is at N_e_ values of a few hundred. As expected, only downstream migrants were detected by genetic analyses, suggesting asymmetric gene flow. This has likely occurred ever since småblank became isolated as a landlocked population and caused the establishment of a source–sink metapopulation structure. This is expected in fast-flowing rivers where it is easier to migrate downstream than upstream (Kawecki and Holt 2002).

Our expectation that genetic variation was lower in the upper (F, G) than in the lower (A–D) subpopulations was not confirmed. Neither had the relative level of genetic variation in the upper and lower subpopulations changed over the last six decades, since the 1950s. The genetic variation in the source population seems not to be declining, and it is not lower than the sink populations, in spite of the asymmetric downstream gene flow. This may indicate an effective source population size (N_e_) large enough to prevent loss of genetic variation in a short period of time (since the 1950s), and larger than the effective sink population size. It should be noted that the area represented by the G samples (the River Mellingselva tributary) is in a more or less pristine condition in terms of human encroachment. The time since the 1950s, corresponding to 8–12 småblank generations, may also have been too short for detecting loss of genetic variation. Moreover, we cannot exclude the possibility that småblank had already been impacted by hydropower development when the first samples were collected in 1955–1956 and 1978–1979. The first hydropower development to influence a major part of River Namsen was the damming of the Namsvatn reservoir (the source of River Namsen) in 1952. Both during the construction and the operational phase, this would have impacted on a major part of the småblank habitat. The weirs in the småblank section of River Namsen were constructed in 1965/1966, 1978, and 1998 (Thorstad et al. 2009).

The subpopulations of småblank are genetically divergent, but our data indicate that they over the last decades have become more similar. This might be caused by an increase in asymmetrical gene flow where downstream movement has become more frequent relative to upstream movement. Whether a downstream sink population depends on the upstream source population for its persistence depends, i.a., on the effective population size of the sink population and different habitat qualities between the source and the sink population (Kawecki and Holt 2002). The construction of weirs has likely impacted more on upstream than on downstream movement rates, thereby increasing the asymmetry of the gene flow. The modifications of the river habitat have been more extensive in the lower part of the distribution area.

The genetic division in subpopulations is probably an advantage to the conservation of genetic variation in småblank, because a number of subpopulations with limited gene flow between them may conserve more genetic variation than a similar number of individuals in one continuous population (Hanski 1999; Tufto and Hindar 2003; Schindler et al. 2010). This is true as long as the effective population size is large enough, and limited movement and gene flow are possible between subpopulations to prevent them from extinction. Human activities leading to fragmentation of the småblank habitat may have reduced or even terminated gene flow among subpopulations. Such fragmentation is expected to cause increased loss of genetic variation, increased inbreeding, and increased risk of extinction (Hanski 1999; Morita and Yamamoto 2002; Hänfling and Weetman 2006). To maintain a healthy metapopulation of småblank, measures should be taken to maintain a natural pattern of two-way movement among subpopulations. Further construction of weirs and other movement barriers is not recommended.

More than half of the distribution area of småblank in the main river has over the last decades been converted from lotic to lentic habitat due to the construction of dams and weirs. The weirs are low dams constructed in order to increase water-covered areas at reduced water flows, largely for esthetic purposes and to improve access to trout fishing. The dams and weirs contribute to remove the stony habitats with fast current used by småblank (Norum 2010), which seem to have similar habitat requirements as juvenile anadromous Atlantic salmon (cf. Heggenes et al. 1999). The fact that about 60% of the annual water volume has been diverted from the upper part of the main river contributes to a reduced annual flow and reduced water velocities. This impacts approximately 53 km, or more than 70%, of the section of the main river inhabited by allopatric småblank. In addition to the increased area with lentic characteristics, the regulated and stabilized water flow of the main river probably contributes to sediment packing of the stony substratum and reduced shelter availability (Finstad et al. 2007). Hence, tributaries may presently become more important as habitat for småblank, as they are less impacted by anthropogenic activities. However, the area available to småblank in the tributaries is restricted to only 2.1 km^2^ (Norum 2010). More detailed sampling is required to provide a better understanding of the fine-scale population structure and to identify the most important areas for småblank.

Many of the European landlocked salmon populations have been subject to heavy pressures due to anthropogenic activities (Berg 1985; Barlaup et al. 2005; Ozerov et al. 2012). Although this is also the case for småblank, our hypothesis that these anthropogenic changes were reflected in a loss of genetic variation (heterozygosity and allelic richness) over the last five to seven decades was not supported.

Four features are usually involved in the concept of rarity: number of individuals and populations, geographical distribution, ecological divergence (habitat specificity), and genetic divergence (Groom et al. 2006). Contrary to the river-resident Atlantic salmon in North America (Scott and Crossman 1973), which occur in several river basins (Webb et al. 2007), småblank is the only one in Europe. The river-resident populations in North America belong to another genetic subgroup than småblank (Lubieniecki et al. 2010; Bourret et al. 2013). Småblank clearly represent a unique genetic cluster, in line with the concept of Evolutionary Significant Unit (ESU), because it is matched by no other Atlantic salmon population, neither in biologic nor genetic characteristics (Waples 1991; Ford 2004).

In conclusion, our results showed a relatively low genetic variation within småblank compared with anadromous Atlantic salmon, but still we found a clear genetic subpopulation structure. Hence, our first hypothesis was partly supported. The observed genetic variation and the complete isolation make småblank an endemic island population. Because the extinction risk is higher in island than mainland populations (Frankham 1997), special care is needed in the management to conserve such populations.

A downstream asymmetric gene flow was recorded within småblank, but the hypothesis predicting a larger genetic variation in the lower compared with the upper subpopulations was not supported. This may indicate that populations in the pristine habitat in the upper section of the småblank area act as a source population, while the populations in the lower section are dependent on the upper populations to maintain their genetic variation.

Our third hypothesis was not supported, as we found no reduction in genetic variation in småblank over the last 50–60 years (12–15 generations). However, we observed a reduced differentiation among the subpopulations within the distribution area.

A major reason for the apparent genetic stability of småblank is likely that its metapopulation structure is still relatively intact. To reduce the risk of extinction and ensure the viability of this endemic form of Atlantic salmon, measures causing further fragmentation of the population should be avoided.

## Data Accessibility

Microsatellite data information: doi:10.5061/dryad.jk42p

## Appendix 1. Appendix Summary statistics for eight microsatellite loci for one anadromous Atlantic salmon population and eight sampling localities of the landlocked Atlantic salmon in the River Namsen watercourse in Norway

**Table:**

Locus	Ssa289	SsOSL438	SsOSL85	Ssa14	Ssa171	u20.19	Ssa197	Ssa408
ANA_78_								
*N*	35	32	30	35	35	31	35	35
#A	4	11	8	2	10	3	19	20
A_R_	3.540	5.470	5.294	1.987	5.797	2.432	7.370	7.681
H_O_	0.514	0.781	0.800	0.543	0.857	0.323	0.971	0.943
H_E_	0.663	0.806	0.815	0.441	0.850	0.362	0.914	0.921
P_H–W_	0.153	0.131	0.268	0.173	0.985	0.718	0.561	0.797
A								
*N*	14	14	14	14	14	14	14	14
#A	2	2	2	1	3	1	4	8
A_R_	1.993	1.595	1.913	1.000	2.959	1.000	3.432	4.880
H_O_	0.357	0.143	0.214	0.000	0.429	0.000	0.571	0.714
H_E_	0.436	0.133	0.293	0.000	0.640	0.000	0.643	0.727
P_H–W_	0.498	0.773	0.313	na	0.177	na	0.326	0.801
B								
*N*	16	16	16	16	16	11	16	16
#A	2	1	2	1	3	1	5	8
A_R_	1.982	1.000	1.970	1.000	2.689	1.000	3.418	4.906
H_O_	0.313	0.000	0.500	0.000	0.625	0.000	0.625	0.688
H_E_	0.404	0.000	0.375	0.000	0.580	0.000	0.650	0.756
P_H–W_	0.364	na	0.182	na	0.903	na	0.931	0.820
C								
*N*	52	49	46	52	52	5	52	51
#A	2	2	2	1	4	1	6	10
A_R_	1.992	1.678	1.868	1.000	2.883	1.000	3.922	4.866
H_O_	0.346	0.163	0.304	0.000	0.654	0.000	0.673	0.627
H_E_	0.464	0.183	0.287	0.000	0.599	0.000	0.719	0.761
P_H–W_	0.067	0.445	0.688	na	0.673	na	0.700	**0.003**
D								
*N*	17	17	17	17	17	11	17	17
#A	2	2	3	1	3	1	6	10
A_R_	1.985	1.936	2.194	1.000	2.747	1.000	3.792	5.576
H_O_	0.588	0.412	0.294	0.000	0.412	0.000	0.647	0.941
H_E_	0.415	0.327	0.337	0.000	0.490	0.000	0.690	0.780
P_H–W_	0.086	0.285	0.851	na	0.067	na	0.951	0.827
E								
*N*	26	24	24	26	26	24	26	26
#A	3	2	2	1	5	1	6	10
A_R_	2.440	1.208	1.947	1.000	3.326	1.000	2.926	5.727
H_O_	0.500	0.042	0.458	0.000	0.923	0.000	0.577	0.846
H_E_	0.455	0.041	0.353	0.000	0.679	0.000	0.589	0.839
P_H–W_	0.910	0.917	0.145	na	0.100	na	0.981	0.410
F								
*N*	28	28	28	28	28	28	28	28
#A	3	2	3	1	5	1	7	9
A_R_	2.164	1.328	2.238	1.000	3.522	1.000	4.086	5.433
H_O_	0.321	0.071	0.393	0.000	0.821	0.000	0.571	0.821
H_E_	0.281	0.069	0.371	0.000	0.682	0.000	0.668	0.820
P_H–W_	0.795	0.845	0.890	na	0.443	na	**0.024**	0.925
G								
*N*	23	23	23	23	23	23	23	23
#A	4	2	2	1	3	1	5	9
A_R_	2.334	1.217	1.792	1.000	2.957	1.000	3.141	5.149
H_O_	0.261	0.043	0.174	0.000	0.739	0.000	0.478	0.826
H_E_	0.303	0.043	0.227	0.000	0.658	0.000	0.546	0.781
P_H–W_	**0.009**	0.915	0.263	na	0.346	na	**0.028**	**0.002**
E_55_								
*N*	15	14	13	15	12	14	15	11
#A	2	1	2	1	3	1	5	7
A_R_	1.719	1.000	1.983	1.000	2.943	1.000	4.239	5.612
H_O_	0.200	0.000	0.538	0.000	0.917	0.000	1.000	1.000
H_E_	0.180	0.000	0.393	0.000	0.622	0.000	0.731	0.826
P_H–W_	0.667	na	0.184	na	0.127	na	**0.017**	**0.001**
E_78_								
*N*	30	26	16	24	19	30	30	11
#A	3	2	2	1	4	1	7	6
A_R_	2.217	1.481	1.918	1.000	3.119	1.000	3.890	5.108
H_O_	0.367	0.115	0.250	0.000	0.579	0.000	0.800	0.818
H_E_	0.313	0.109	0.305	0.000	0.633	0.000	0.687	0.793
P_H–W_	0.680	0.755	0.473	na	0.821	na	0.832	0.569
G_56_								
*N*	10	10	8	10	10	10	10	10
#A	1	1	2	1	3	1	4	5
A_R_	1.000	1.000	2.000	1.000	2.889	1.000	3.510	4.236
H_O_	0.000	0.000	0.625	0.000	0.600	0.000	0.500	0.700
H_E_	0.000	0.000	0.492	0.000	0.585	0.000	0.615	0.745
P_H–W_	na	na	0.445	na	0.951	na	0.320	0.929
G_78_								
*N*	19	19	15	19	18	19	19	13
#A	3	1	2	1	4	1	6	7
A_R_	2.267	1.000	1.999	1.000	3.594	1.000	3.572	5.174
H_O_	0.368	0.000	0.400	0.000	0.611	0.000	0.579	0.615
H_E_	0.314	0.000	0.480	0.000	0.674	0.000	0.615	0.799
P_H–W_	0.809	na	0.519	na	0.338	na	0.546	0.279
A_79_								
*N*	26	23	13	26	12	23	23	17
#A	4	3	2	1	3	1	6	4
A_R_	2.379	1.609	1.878	1.000	2.859	1.000	3.627	3.280
H_O_	0.615	0.130	0.154	0.000	0.417	0.000	0.652	0.412
H_E_	0.510	0.124	0.260	0.000	0.538	0.000	0.601	0.538
P_H–W_	0.704	0.990	0.140	na	0.052	na	0.459	0.163

*N* is sample size, #A is number of alleles, A_R_ is allelic richness, H_O_ is observed heterozygosity, H_E_ is expected heterozygosity, P_H–W_ is probability of conformance to Hardy–Weinberg expectation. Sampling localities are shown in Table 1 and Fig. 1. *P*-values < 0.05 are in bold.

## Appendix 2. Pairwise *F*_ST_ (lower triangle) and *P*-values (upper triangle) between eight sampling localities of landlocked Atlantic salmon and one sample of anadromous Atlantic salmon from the River Namsen watercourse. Significant after corrections for multiple testing

**Table:**

Pop	ANA78	A	B	C	D	E	F	G	E_55_	E_78_	G_56_	G_78_	A_79_
ANA78		**∼0**	**∼0**	**∼0**	**∼0**	**∼0**	**∼0**	**∼0**	**∼0**	**∼0**	**∼0**	**∼0**	**∼0**
A	0.216		0.615	0.889	0.891	0.020	**∼0**	**∼0**	0.001	0.016	**∼0**	**∼0**	0.940
B	0.233	−0.006		0.338	0.090	0.083	**∼0**	**∼0**	**0.000**	0.326	**∼0**	**∼0**	0.227
C	0.260	−0.012	0.003		0.713	**∼0**	**∼0**	**∼0**	**0.000**	**0.000**	**∼0**	**∼0**	0.462
D	0.217	−0.008	0.026	−0.003		0.001	**∼0**	**∼0**	0.001	0.003	**0.000**	**∼0**	0.480
E	0.228	0.018	0.020	0.040	0.046		0.087	0.021	**∼0**	0.360	0.038	0.033	**∼0**
F	0.236	0.060	0.055	0.086	0.082	0.017		0.483	0.018	0.204	0.114	0.052	**∼0**
G	0.254	0.077	0.061	0.095	0.098	0.022	−0.003		**∼0**	0.244	0.106	0.171	**∼0**
E_55_	0.225	0.054	0.062	0.073	0.075	0.048	0.019	0.045		0.002	0.001	**∼0**	**0.000**
E_78_	0.243	0.035	0.005	0.045	0.060	0.011	0.013	0.011	0.040		0.035	0.076	**0.000**
G_56_	0.227	0.092	0.078	0.109	0.094	0.036	0.020	0.032	0.051	0.034		0.059	**∼0**
G_78_	0.224	0.065	0.049	0.083	0.080	0.019	0.010	0.014	0.045	0.019	−0.001		**∼0**
A_79_	0.251	−0.010	0.030	0.007	0.008	0.062	0.116	0.137	0.118	0.084	0.151	0.112	

Sampling localities are shown in Table 1 and Fig. 1.

*P*-values < 0.05 are in bold.
